# Mitochondrial DNA mutations in renal cell carcinomas revealed no general impact on energy metabolism

**DOI:** 10.1038/sj.bjc.6602929

**Published:** 2006-01-10

**Authors:** D Meierhofer, J A Mayr, K Fink, N Schmeller, B Kofler, W Sperl

**Affiliations:** 1 Department of Paediatrics, Paracelsus Private Medical University Salzburg, Muellner Hauptstr. 48, A-5020 Salzburg, Austria; 2 Department of Urology, Paracelsus Private Medical University Salzburg, Muellner Hauptstr. 48, A-5020 Salzburg, Austria

**Keywords:** oxidative phosphorylation, mitochondrial encephalomyopathy with lactic acidosis and stroke-like episodes, denaturing HPLC, mitochondrial DNA mutations, renal cell carcinoma

## Abstract

Previously, renal cell carcinoma tissues were reported to display a marked reduction of components of the respiratory chain. To elucidate a possible relationship between tumourigenesis and alterations of oxidative phosphorylation, we screened for mutations of the mitochondrial DNA (mtDNA) in renal carcinoma tissues and patient-matched normal kidney cortex. Seven of the 15 samples investigated revealed at least one somatic heteroplasmic mutation as determined by denaturating HPLC analysis (DHPLC). No homoplasmic somatic mutations were observed. Actually, half of the mutations presented a level of heteroplasmy below 25%, which could be easily overlooked by automated sequence analysis. The somatic mutations included four known D-loop mutations, four so far unreported mutations in ribosomal genes, one synonymous change in the ND4 gene and four nonsynonymous base changes in the ND2, COI, ND5 and ND4L genes. One renal cell carcinoma tissue showed a somatic A3243G mutation, which is a known frequent cause of MELAS syndrome (mitochondrial encephalomyopathy, lactic acidosis, stroke-like episode) and specific compensatory alterations of enzyme activities of the respiratory chain in the tumour tissue. No difference between histopathology and clinical progression compared to the other tumour tissues was observed. In conclusion, the low abundance as well as the frequently observed low level of heteroplasmy of somatic mtDNA mutations indicates that the decreased aerobic energy capacity in tumour tissue seems to be mediated by a general nuclear regulated mechanism.

Renal cell carcinoma is the most common malignancy arising in the adult kidney. Already five decades ago it was shown that the vast majority of tumours display a high rate of glycolysis under aerobic conditions (Warburg, 1956). Accordingly, in renal cell carcinomas, an increase of proteins involved in major steps of the glycolytic pathway and a decrease of the gluconeogenic reactions was observed (Faure Vigny *et al*, 1996; Unwin *et al*, 2003). Furthermore, a depletion of the activity of several mitochondrial enzymes was demonstrated (Faure Vigny *et al*, 1996; Unwin *et al*, 2003; Meierhofer *et al*, 2004). Human solid tumours endure profound hypoxia, which indicates that adaptation to hypoxic conditions is a crucial step in tumour progression. Recently, it was postulated that deficiency of the von Hippel Lindau (VHL) protein, which is observed in most renal carcinomas, could be one of the factors responsible for downregulation of the biogenesis of complexes of the oxidative phosphorylation (OXPHOS) (Shiao *et al*, 2000; Hervouet *et al*, 2005).

Since some subunits of the OXPHOS are encoded by mitochondrial DNA (mtDNA), alterations of mtDNA may influence OXPHOS activity. Mitochondrial DNA has a high mutation rate due to the damage produced by free radicals, the lack of protective action by histones and the limited capacity of repair of the mtDNA (Brown *et al*, 1979; Pettepher *et al*, 1991). The mutation rate of mtDNA has been reported to be as much as two orders of magnitude greater than that of nuclear DNA (Khrapko *et al*, 1997). A mutation in mtDNA expands either partially (heteroplasmy) or totally replaces all mtDNA (homoplasmy). However, it is still unclear how mutated mtDNA expands in cells.

Recently, a high incidence of specific mtDNA alterations has been reported for gastric (Maximo *et al*, 2001; Wu *et al*, 2005), prostate (Jeronimo *et al*, 2001; Petros *et al*, 2005), pancreatic (Jones *et al*, 2001), skin (Girald-Rosa *et al*, 2005), colorectal (Polyak *et al*, 1998; Hibi *et al*, 2001a; Lievre *et al*, 2005), urinary bladder (Fliss *et al*, 2000), thyroid (Yeh *et al*, 2000), oesophageal (Hibi *et al*, 2001b; Kumimoto *et al*, 2004), liver (Nishikawa *et al*, 2001), breast (Richard *et al*, 2000; Tan *et al*, 2002; Zhu *et al*, 2005), uterine cancers (Pejovic *et al*, 2004) as well as chromophobe renal cell carcinoma (Nagy *et al*, 2002). Of all mtDNA mutations reported in cancer tissues, only a few are known to be of pathological relevance as shown for patients with disorders of the mitochondrial energy metabolism. For example, a G5521A mutation, which is known to cause a late onset mitochondrial myopathy (Silvestri *et al*, 1998), was detected in a lung cancer tissue (Fliss *et al*, 2000). A mutation (G13708A) reported in patients with Leber's hereditary optic neuropathy (LOHN) (Brown *et al*, 1992) was found in a breast cancer tissue (Parrella *et al*, 2001) and a mutation typical for mitochondrial encephalomyopathy with lactic acidosis and stroke-like episodes (MELAS) was detected in a colon cancer sample (Lorenc *et al*, 2003). As would be anticipated, these patients, who all were carrying only tumour-specific pathogenic mtDNA mutations, did not show clinical signs of mtDNA-related disease. Recently, it was shown that the introduction of a pathogenic mtDNA mutations, for example, in the mitochondrial-encoded ATPase 6 gene (MTATP6), can lead to declined respiration and accelerated growth of tumour cells via inhibition of apoptosis (Petros *et al*, 2005; Shidara *et al*, 2005).

To determine whether the downregulation of the enzyme activities reported in renal carcinoma is associated with mtDNA mutations, we screened the entire mtDNA of 15 paired tumour and the corresponding normal kidney samples by denaturating HPLC analysis (DHPLC) analysis. Occurrence and type of somatic mtDNA mutations were compared to the enzymatic activity of respiratory chain complexes.

## MATERIALS AND METHODS

### Patients

Tumour and the corresponding healthy cortex tissue from 15 patients were obtained by nephrectomy at the Department of Urology, Salzburg as previously reported (Meierhofer *et al*, 2004). The tumour classification was performed according to Storkel *et al* (1997). DNA was isolated by proteinase K digestion followed by phenol/chloroform extraction.

### PCR amplification of entire mitochondrial genome and mutation analysis

The whole human mtDNA of all 15 patients was amplified using 48 overlapping PCR fragments. Mutation detection by DHPLC was performed as reported previously (Meierhofer *et al*, 2005). As estimated from the areas under the peaks of the DHPLC chromatogram, the degree of heteroplasmy was divided into lower 25% (<25%), between 25 and 75% (25–75%) and over 75% (>75%). All samples showing a homo- and heteroduplex peak of the same extent in DHPLC analysis were sequenced directly. Heteroplasmic peaks lower than the homoplasmic peak were manually collected and reamplified as previously reported (Meierhofer *et al*, 2005).

Mutations were analysed with the Beckman software investigator (Fullerton, CA, USA). Exact positions of mutations and amino-acid changes were defined with the mitoAnalyser tool (MitoAnalyzer, 2000), using the mtDNA genbank sequence J01415.1 as reference.

### Determination of mutational load of the A3243G mutation

A PCR fragment was amplified with the following primers: forward, 5′-TCCCTGTACGAAAGGACAAGA-3′; reverse, 5′-AGGAGTAGGAGGTTGGCCAT-3′ and with cycling and PCR conditions as reported previously (Meierhofer *et al*, 2005). The last PCR cycle was followed by a denaturating and reannealing step. Restriction digestion with *Hae*III, which digests only the mutation-specific restriction side 3243G and one control restriction side on each side of the PCR fragment, was performed. The percentage of mutational load was detected by densitometry of ethidium-bromide-stained agarose gels (Uziel *et al*, 1997; van den Bosch *et al*, 2004).

### Enzyme measurements

The following enzyme activities: citrate synthase, complex II, complex IV and oligomycin-sensitive ATPase activity of complex V were determined and reported previously (Meierhofer *et al*, 2004). In addition, complex I activity was measured according to Rustin *et al* (1994).

### Western blot analysis of the VHL tumour suppressor protein

After separation of the 600 g homogenate (Meierhofer *et al*, 2004), 18 *μ*g protein per lane were loaded on a 10% polyacrylamide gel and Western blot analysis was performed according to Berger *et al* (2003). The following antibodies were used: mouse monoclonal antibody against VHL protein (Cat# 556347; BD Bioscience, Palo Alto, CA, USA; 1 *μ*g/ml), alkaline phosphatase-conjugated rabbit anti-mouse immunoglobulins (Dako, Golstrup, Denmark; 1 : 5000).

## RESULTS

### MtDNA mutations

**Figure 1:**
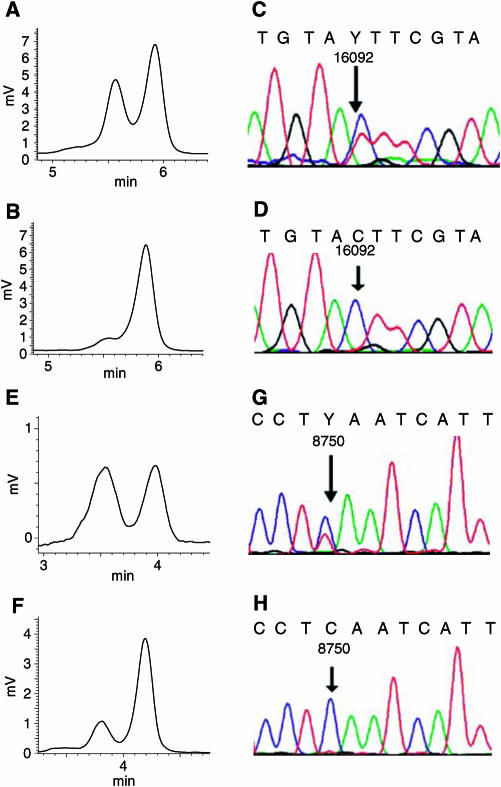
Analysis of a shift of heteroplasmy in matched tissue pairs. Denaturating HPLC analysis of a PCR fragment (15587–16185) from case 11 at 56.0°C oven temperature (**A**, **B**). Denaturating HPLC analysis revealed a heteroplasmy of the kidney tissue of about 40% (**A**) and of the corresponding renal carcinoma tissue of over 95% (**B**). Sequence analysis indicates a 16092C/T heteroplasmy in the kidney tissue (**C**) and the 16092C mutation in the corresponding renal carcinoma (**D**). Denaturating HPLC analysis of a PCR fragment (8466–8925) from case 12 at 57.8°C oven temperature (**E**, **F**). The kidney tissue shows a heteroplasmy of about 50% (**E**) and the corresponding carcinoma tissue of about 80% (**F**). Sequence analysis revealed a 8750C/T heteroplasmy in the kidney tissue (**G**) and the 8750C variation in the corresponding carcinoma tissue (**H**). Y=C+T.

The mutation screening of the entire mtDNA of 15 primary renal carcinoma and matched control kidney cortex tissues was carried out by DHPLC analysis. Five matched tumour/kidney pairs showed one to two heteroplasmic mutations (six D-loop, one 16s rRNA and one COI) of the mtDNA with no difference of the degree of heteroplasmy in the two tissues (Table 1). Interestingly, in two patients, a shift in heteroplasmy from the kidney to the corresponding tumour tissue was observed (cases 11, 12: Table 1; Figure 1). Loss of low-level heteroplasmy present in normal cortex tissue was observed in cases 4 (310 C insertion; A8483G) and 14 (T72C). Only three conventional renal carcinomas with grades 1, 2 and 4 carried neither a somatic nor a heteroplasmic mtDNA mutation.

In total, 14 types of somatic genetic mutations were detected in seven patients (Table 2). No somatic homoplasmic mutations were detected in any of these 15 renal carcinoma tissues. Notably, the level of heteroplasmy of somatic mutations was frequently below 25% (Table 2), whereas all nonsomatic heteroplasmies were above 25% (Table 1).

**Figure 2:**
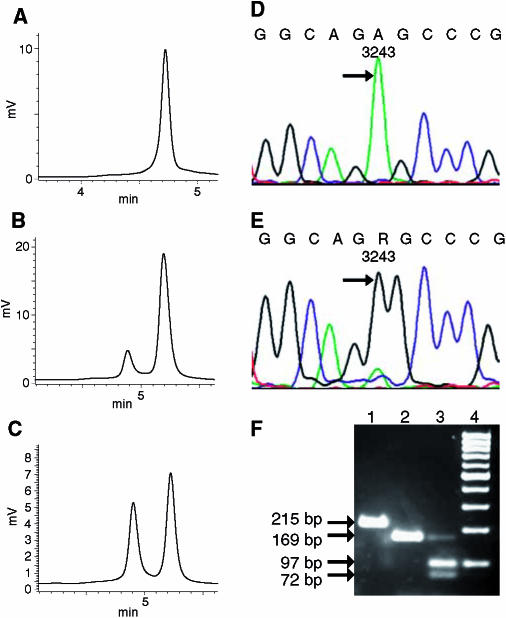
Mitochondrial DNA analysis of the renal carcinoma with the somatic A3243G mutation. Denaturating HPLC analysis of a PCR fragment (3079–3505) of case 4 at 58°C oven temperature (**A**–**C**). The kidney tissue shows a single homoplasmic peak (**A**), the corresponding renal carcinoma tissue a heteroplasmy over 90% (**B**) and the mixture of denaturated and reannealed PCR product of kidney and the corresponding tumour tissue resulted in one hetero- and homoduplex peak of the similar height (**C**). Sequence analysis of the PCR product indicated the wild-type 3243A variant in the unaffected kidney tissue (**D**), and the 3243G mutation in the corresponding carcinoma tissue (**E**), as indicated by arrows. Agarose gel analysis of a restriction digestion of a PCR fragment (3118–3332) with *Hae*III, which specifically recognizes the 3243G mutation, and two control sites within the PCR fragment yielding two small fragments (**F**): undigested full-length PCR fragment of 215 base pairs (lane 1); kidney tissue resulting in a 169-base pair fragment (lane 2); carcinoma tissue resulting in a weak 169-base pair fragment of the residual wild-type 3243A variant as well as 72- and 97-base pair fragments, indicating the 3243G mutation (lane 3); 100 bp molecular weight marker (lane 4). R=G+A.

The somatic mutations included four known D-loop mutations, four so far unreported mutations in ribosomal genes, one synonymous change in the ND4 gene and four nonsynonymous base changes in the ND2, COI, ND5 and ND4L genes (Table 2). Interestingly, in one tumour tissue, the tRNA^LEU(UUR)^ gene revealed a A to G transition at position 3243, which is a known frequent cause of MELAS syndrome (Figure 2). Restriction digestion with *Hae*III of the PCR fragment containing the A3243G mutation revealed an 89% mutational load (Figure 2F). The corresponding kidney tissue was unaffected.

Furthermore, the known MELAS suppresser G12300A mutation (El Meziane *et al*, 1998) was not present in the tumour sample with the A3243G mutation.

### Enzyme activities

Biochemical analysis of OXPHOS and Krebs-cycle enzyme activities of 37 renal carcinoma tissues were reported in our previous study (Meierhofer *et al*, 2004), which included the 15 patients presented here (Table 3). A total of 14 renal carcinoma tumour tissues, investigated in this study, showed a depletion of the mtDNA content (46%) combined with a decreased activity of complex I (16%), complex II (23%), complex IV (29%), oligomycin-sensitive ATPase activity of complex V (25%) and citrate synthase (72%) compared to matched normal cortex tissues (Table 3) (Meierhofer *et al*, 2004).

This is in clear contrast to the tumour tissue with the A3243G mutation, which showed an upregulation of the mtDNA content (170%) and increased enzyme activities of complex II (138%), oligomycin-sensitive ATPase activity of complex V (1530%) and citrate synthase (575%) and a downregulation of the mitochondrial- and nuclear-encoded enzyme activities of complex I (22%) and complex IV (78%) related to the matching control tissue (Table 3).

One tumour tissue displayed only an elevated level of citrate synthase (300%). As expected, no pathogenic mutation was found in the mtDNA of this sample.

### Western blot analysis of the VHL tumour suppressor protein

**Figure 3:**
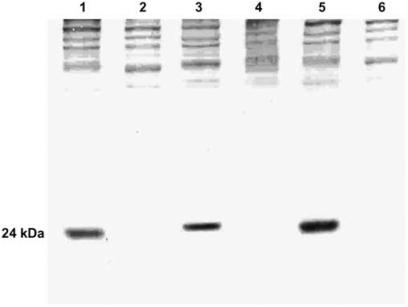
Western blot analysis of VHL protein content in extracts of three matched tissue pairs. Case 3: kidney control tissue (lane 1) and renal carcinoma tissue (lane 2); case 4: kidney control tissue (lane 3) and renal carcinoma tissue with the somatic A3243G mutation (lane 4); case 5: kidney control tissue (lane 5) and renal carcinoma tissue (lane 6); VHL: 24 kDa.

The von Hippel-Lindau tumour suppressor protein (VHL) is frequently absent in renal carcinoma tissues. A recent study has shown that VHL deficiency is one of the factors responsible for downregulation of the biogenesis of OXPHOS complexes in renal carcinoma (Hervouet *et al*, 2005). To exclude that the altered enzyme activities of the OXPHOS complex in the tumour tissue with the A3243G mutation are based on the presence of the VHL protein, we tested tumour tissues by Western blot analysis for VHL content. In the renal carcinoma tissue harbouring the A3243G mutation as well as in the two other renal carcinoma tissues investigated, lack of VHL protein was observed (Figure 3).

## DISCUSSION

In the present study, we evaluated the influence of mtDNA mutations on the OXPHOS capacity of renal carcinoma tissues. The mtDNA was more frequently mutated in renal carcinoma than in kidney cortex tissues. This is in line with somatic mtDNA mutations reported in a wide variety of human neoplasias (Fliss *et al*, 2000; Jones *et al*, 2001; Nagy *et al*, 2002; Tan *et al*, 2002). Somatic mutations in coding regions of the mtDNA in tumour tissues are either silent or mostly nonpathogenic polymorphisms. Only a few pathogenic mtDNA mutations have been reported (Brown *et al*, 1992; Parrella *et al*, 2001; Lorenc *et al*, 2003). However, no biochemical analysis has been performed to evaluate the consequences of these mutations in the tumour tissues. Here, we show the first combined genetic and biochemical analysis of a mitochondrial A3243G mutation in a tumour tissue. The renal carcinoma tissue with the somatic A3243G mutation showed a compensatory upregulation of enzyme complexes, which are only nuclear encoded. This is in clear contrast to the coordinated downregulation of all components necessary for mitochondrial energy metabolism, which we found in 92% of all renal carcinoma tissues irrespective of tumour stage and progression (Meierhofer *et al*, 2004). However, the low activity of the partially mtDNA-encoded complexes of the respiratory chain in the tumour tissue with the somatic A3243G mutation will lead to similar low oxidative capacity as in the other renal cell carcinomas. Accordingly, histology and clinical progression did not differ.

The biochemical consequences of the A3243G mutation in the renal carcinoma tissue are in agreement with the decreased activity of the mitochondrial- and nuclear-encoded complex I and IV reported in muscle biopsy and fibroblasts of patients with MELAS syndrome carrying the A3243G mutation (de Vries *et al*, 1994; Wilichowski *et al*, 1998; Pronicki *et al*, 2002). Our findings indicate that the biochemical changes caused by the pathogenic A3243G mutation are not dependent on tissue type and therefore, it seems to be a general cellular compensatory mechanism.

The discrepancy between the normal enzyme activity of complex II *vs* the decreased complex I and IV enzyme activities in the sample with the A3243G mutation may lie partly in the fact that the latter complexes are involved in proton pumping, whereas the former is not.

Lack of the VHL protein and somatic alterations in the VHL gene are found in about half of conventional renal carcinomas (Brauch *et al*, 2000). Hervouet *et al* (2005) reported that the transfection of the VHL gene in VHL-deficient renal carcinoma cells increases mtDNA and respiratory chain protein contents and permitted the cells to rely on their mitochondrial ATP production to grow in the absence of glucose. Presence of an intact VHL protein in the tumour tissue could have explained the observed upregulation of the enzyme activities in the renal carcinoma tissue with the A3243G mutation. However, the VHL protein was not detectable in our case harboring the A3243G mutation.

In contrast to several other studies reporting mtDNA mutations in cancer tissues, no somatic homoplasmic mutations were detected in the renal samples investigated here. One explanation might be that we used sensitive DHPLC analysis with a detection limit of heteroplasmy of down to 1% and up to 99%, respectively (Meierhofer *et al*, 2005). Furthermore, contaminations of the tumour tissues with blood and epithelial cells of vessels might contribute to a certain degree of heteroplasmy. However, these contaminations might also have been latent in previous studies. Finally, because of high background in sequence analysis, heteroplasmies of over 80% might appear as homoplasmic mutation and therefore, high-level of heteroplasmy in this study might be equivalent to homoplasmy in other studies (Meierhofer *et al*, 2005). In accordance, the heteroplasmy in the tumour tissue of case 12 (C8750T) is only visible in DHPLC analysis but not in direct sequence analysis (Figure 1F and H). For the same reason, low-level heteroplasmy might be overlooked by automated sequence analysis.

It is difficult to compare the frequency of mtDNA mutations in different studies analysing mtDNA mutations in distinct cancer types, because most of these studies only analysed parts of the mitochondrial genome, mostly the D-loop. Complete sequence analysis of the mtDNA of 10 primary ovarian carcinomas revealed somatic mtDNA mutations in 60% of the tumour samples (Liu *et al*, 2001). Another study found that 74% of breast cancer samples had at least one somatic mtDNA mutation (Tan *et al*, 2002), indicating that the incidence of homoplasmic or high level heteroplasmic somatic mutations in these tumours is higher than in renal cell carcinoma reported here.

Mutations in the D-loop regulatory region might alter the rate of DNA replication by modifying the binding affinity of significant trans-activating factors. In the renal carcinomas presented here, only four out of the 14 somatic mutations affected the D-loop. This cannot explain the decrease of the mtDNA content observed in the majority of renal carcinomas (Simonnet *et al*, 2002; Meierhofer *et al*, 2004). In agreement with other studies, either G to A or T to C transitions were observed, which is consistent with the mutagenic spectra of oxidative damage (Lee *et al*, 2004).

In half of the somatic mutations, the level of heteroplasmy was between 1 and 25%. Phenotypic manifestation of a genetic defect of the mtDNA occurs only if a threshold level is exceeded. Although the phenotypic threshold depends on the type of mutation and tissue, it has been shown that a heteroplasmy at least over 60% is necessary to show effects on the enzyme activity of respiratory chain complexes (Rossignol *et al*, 2003). Therefore, a primary role of the low heteroplasmic mutations in downregulation of OXPHOS activity and renal carcinogenesis is unlikely. Accumulation of mtDNA mutations in tumours can be explained without selection (Coller *et al*, 2001). Heteroplasmic mutations might have accumulated in cancer cells by random drift of a pre-existing heteroplasmy (Nekhaeva *et al*, 2002). This random drift will also explain the observed shift of the degree of heteroplasmy of a mixture of mutated and wild-type mtDNA already detectable in tumour progenitor cells.

In conclusion, the low frequency as well as the low level of heteroplasmy of the somatic mtDNA mutations in renal carcinomas does not indicate a major contribution of these alterations in tumour development. Furthermore, the downregulation of the mitochondrial energy metabolism observed in renal carcinomas cannot be explained by the presence of mtDNA mutations. Even, the case carrying the A3243G mutation exhibits normal tumour histology and low overall oxidative capacity. More likely, a general nuclear-encoded mechanism results in the adaptation of the aerobic energy metabolism in renal carcinoma.

## Figures and Tables

**Table 1:** Nonsomatic heteroplasmies in matched tissue pairs

**Case**	**Tumour type**	**Grade**	**Nucleotide position**	**Heteroplasmy^a^ in kidney/tumour (%)**	**Gene**	**Amino acid**	**Notes**
6	Papillary RC	2	A16206G	>75	D-loop		d
6			C16348T	>75	D-loop		d
7	Sarkomatoid RC	4	A16206G	25–75	D-loop		d
7			C16348T	25–75	D-loop		d
11	Conv. RC^b^	3	T16092C	from 40 to >95	D-loop		c
12	Conv. RC	2	T8750C	from 50 to 80	ATP6	L75S	d
13	Conv. RC	1/2	T1809C	>75	16S rRNA		d
13			G6260A	25–75	COI	syn	c
14	Papillary RC	1	G75A	25–75	D-loop		c
15	Conv. RC	1	T16093C	25–75	D-loop		c

a Estimated by denaturating HPLC analysis.

b Conv.=conventional; RC=renal carcinoma; syn=synonymous.

c=Previously reported polymorphism (MITOMAP database).

d=Novel mutation.

**Table 2:** Gain of somatic mutations of the entire mitochodrial DNA genome in renal carcinoma tissues

**Case**	**Tumour type**	**Grade**	**Nucleotide position**	**Heteroplasmy^a^ in tumour (%)**	**Gene**	**Amino acid**	**Notes**
1	Conv. RC^b^	2/3	C338T	<25	D-loop		c
1			A1578G	<25	12S rRNA		d
1			G12007A	<25	ND4	syn	c
2	Conv. RC	3	G4584A	<25	ND2	A39T	d
3	Conv. RC	2	G94A	>75	D-loop		c
3			A7423G	<25	COI	E507G	d
4	Conv. RC	3	T204C	25–75	D-loop		c
4			A3243G	>75	tRNA ^LEU(UUR)^		MELAS
4			G1169A	>75	12S rRNA		d
4			C12510A	>75	ND5	D58E	d
5	Papillary RC	1	T2222C	25–75	16S rRNA		d
6	Papillary RC	2	C1566T	<25	12S rRNA		d
6			T10579C	<25	ND4L	M37T	d
7	Sarkomatoid RC	4	C16174T	25–75	D-loop		c

a Estimated by denaturating HPLC analysis.

b Conv.=conventional; RC=renal carcinoma; syn=synonymous.

^c^Previously reported polymorphism (MITOMAP database).

^d^Novel mutation.

**Table 3:** Median enzyme activities of case 4 with the somatic A3243G mutation in the tumour tissue compared to 14 matched tissue pairs

	**Kidney cortex**			**Renal carcinoma tissue**		
**Enzyme**	**Case 4**	**Median (*n*=14)**	**Range**	**Case 4**	**Median (*n*=14)**	**Range**
CS	63	99	60–161	362	66	27–486
CI	37	50	11–95	8	6	1–17
CII	96	115	31–206	132	22	6–51
CIV	97	181	44–278	76	36	21–108
CV	27	48	28–125	412	13	0–63

Values in U per g protein.

RC=renal carcinoma; CS=citrate synthase; CI=complex I; CII=complex II; CIV=complex IV; CV=complex V.
